# Ten Years of Lateral Flow Immunoassay Technique Applications: Trends, Challenges and Future Perspectives

**DOI:** 10.3390/s21155185

**Published:** 2021-07-30

**Authors:** Fabio Di Nardo, Matteo Chiarello, Simone Cavalera, Claudio Baggiani, Laura Anfossi

**Affiliations:** Department of Chemistry, University of Torino, 10125 Torino, Italy; matteo.chiarello@unito.it (M.C.); simone.cavalera@unito.it (S.C.); claudio.baggiani@unito.it (C.B.); laura.anfossi@unito.it (L.A.)

**Keywords:** lateral flow immunoassay, lateral flow assay applications, paper-based biosensor, immunochromatographic strip test, rapid diagnostic test, point-of-care testing

## Abstract

The Lateral Flow Immunoassay (LFIA) is by far one of the most successful analytical platforms to perform the on-site detection of target substances. LFIA can be considered as a sort of lab-in-a-hand and, together with other point-of-need tests, has represented a paradigm shift from sample-to-lab to lab-to-sample aiming to improve decision making and turnaround time. The features of LFIAs made them a very attractive tool in clinical diagnostic where they can improve patient care by enabling more prompt diagnosis and treatment decisions. The rapidity, simplicity, relative cost-effectiveness, and the possibility to be used by nonskilled personnel contributed to the wide acceptance of LFIAs. As a consequence, from the detection of molecules, organisms, and (bio)markers for clinical purposes, the LFIA application has been rapidly extended to other fields, including food and feed safety, veterinary medicine, environmental control, and many others. This review aims to provide readers with a 10-years overview of applications, outlining the trends for the main application fields and the relative compounded annual growth rates. Moreover, future perspectives and challenges are discussed.

## 1. Introduction

Conventional laboratory-based analytical methods like high-performance liquid chromatography (HPLC), gas chromatography (GC), mass spectrometry (MS), enzyme-linked immunosorbent assay (ELISA) and real time polymerase chain reaction (qPCR) usually require complex and long procedures to obtain a result [1,2,3], but several situations often require a fast and on-site analyte detection [4,5]. As a consequence, in the last decades, the scientific research has been focused more and more on the development and optimization of portable, affordable, and user-friendly rapid methods of analysis for point-of-need (PON) testing [6,7,8].

Immunochemical bioanalytical methods represent one of the most versatile strategies for point-of-need applications thanks to their ability to give highly specific and sensitive results [9]. It is not by chance that most screening and rapid methods are based on immunoassays. An immunoassay is a biochemical test that is commonly used to measure the concentration of target molecules. This method is based on the reaction of an analyte/antigen (Ag) with a selective antibody (Ab) forming an Ab–Ag complex. The efficacy of immunoassay is mainly based on the efficiency of Ab–Ag complex formation and on the ability to detect the rate of complex formation.

Among the different immunoassay-based analytical platforms, the lateral flow immunoassay technique (LFIA), also known as immunochromatographic strip test (ICST), or rapid diagnostic test (RDT), has become one of the most successful analytical platforms for decentralized or point-of-need testing strategy requiring little to no supporting infrastructure. The LFIA is a paper-based (bio)analytical technique for the on-site detection of target substances, where the sample is added on a standalone device and the result is obtained in a few minutes. LFIAs satisfied all the criteria of an ideal POCT that is required to be “ASSURED” (Affordable, Sensitive, Specific, User-friendly, Rapid and robust, Equipment-free, and Delivered) [10]. Initially referred to diagnostic tests for sexually transmitted infections, the ASSURED criteria have become the benchmark for any point of care tests (POCT) and more in general for any point of need tests.

The LFIAs success can be also noticed also considering their position in the commercial landscape. In fact, in the 2019 the global market for lateral flow tests was estimated at about US $5.98 billion and it is projected to reach US $10.36 billion by 2027, growing at a compounded annual growth rate (CAGR) of 7.7% from 2020 to 2027 [11].

Thanks to their advantages of simplicity, rapidity, cost-effectiveness and no requirement of equipment or technical expertise, the LFIAs are very useful especially in low-resource field environments and in developing countries that cannot afford standard instrumentation to perform analyses. However, they are widely used also in developed countries with the aim of increasing the number of analyses, reaching high-throughput goals and rapid decision-making in several fields, while mitigating costs. For example, in 2019, North America and Europe dominated the global lateral flow assay market holding the majority shares [11].

**Figure 1:**
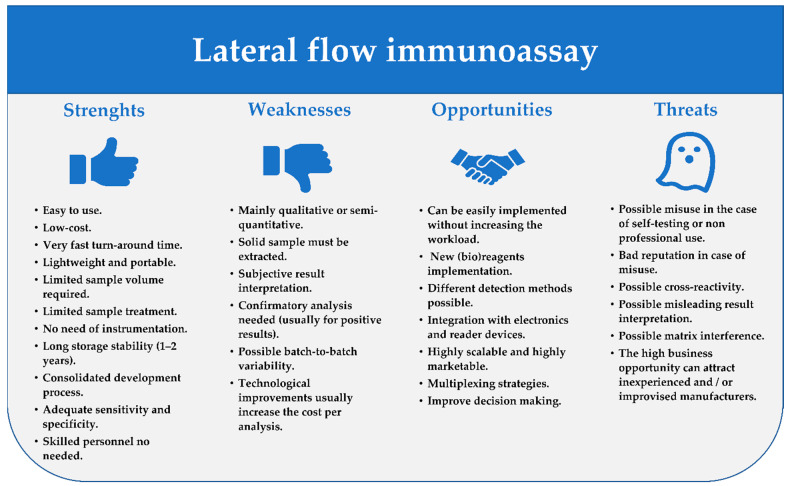
SWOT analysis of the LFIA technique considering its inherent features.

The LFIA can be considered as a sort of lab-in-a-hand and together with other PON tests has represented a paradigm shift from sample-to-lab to lab-to-sample aiming to improve decision making and turnaround time [4]. The very attractive features of LFIA have driven to reach a wide acceptance and appeal of this technique (the main LFIA features are reported in Figure 1). Therefore, from the detection of hormones, parasites, bacteria, cells, viruses, biological markers for clinical purposes, the LFIA application has been rapidly extended to other fields, including food and feed safety, veterinary medicine, environmental control, forensic analysis, and many others [12,13,14].

Even if much innovation has been devoted towards the LFIA sensitivity enhancement [15,16,17], most of the developed LFIA have more than adequate sensitivity for the detection of the most different analytes. Usually, both for the competitive and the non-competitive format, the LFIA limit of detections are between 0.1 and 10 ng mL^−1^ and very rarely they fall below 0.1 ng mL^−1^ [4,14,17]. The adequate analytical sensitivity, together with versatility and good usability, made LFIA the most commercially available POC diagnostic format [13]. This is also due to the fact that in contrast to other promising paper-based analytical platforms that mainly remain as laboratory prototypes, LFIA devices easily enter into real-life applications with a high market penetration mainly because they do not need extensive upgrades to become an end-user device [18]. The wide acceptance of LFIA devices also played an eminent role in the use of the platform for the detection of the severe acute respiratory syndrome coronavirus 2 (SARS-CoV-2), in the current pandemic situation, as we can still notice and as extensively reported in the literature [19,20,21,22,23,24,25,26,27,28].

Previous reviews about LFIA have provided an overview on specific topics such as the use of particular labels [29], or the use of particular molecular recognition element [30,31], sensitivity enhancement and instrumental detection methods [15,16,17], multiplex approach [32,33], and general improvements [34].

The aim of this review is to analyse the lateral flow assay applications state-of-the-art within the 2010–2019 period, providing the respective trends. The year 2020 has not been included because some articles may be not yet indexed in the database. Moreover, the year 2020 could be a source of bias for the trend of clinical application field, due to plenty of publications related to the detection of the SARS-CoV-2. Nonetheless, some considerations regarding the potential opportunities for LFIA devices arose from the pandemic situation will be provided, together with an overview of the most recent and hot applications for each relevant field (not fully comprehensive) mainly covering the 2020–2021 time frame.

In more details, in the following sections the general principles and the fundamental components of LFIAs are briefly introduced. Then, we introduce the literature review methodology and the most relevant LFIA application fields, i.e., clinical, food safety, veterinary, environmental, and others, underlying the LFIA benefits in the respective fields. Subsequently, we report the trends and the CAGR for the main application fields, considering the multiplexing aspect as well. We also discuss what we think is essential to foster an increasing implementation of LFIAs, focusing on the critical challenges to be addressed. Finally, our perceptions regarding the future development of LFIA devices is provided.

## 2. General Principles and the Fundamental Components of LFIAs

**Figure 2:**
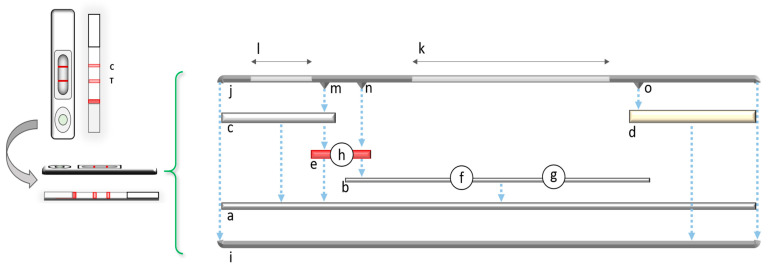
The structure of a typical LFIA strip. (**a**) backing support, (**b**) nitrocellulose porous membrane (**c**) sample pad, (**d**) absorbent pad, (**e**) conjugate pad, (**f**,**g**) immunoreagents areas, (**h**) labelled immunoreagent, (**i**,**j**) cassette, (**k**) reading window, (**l**) sample well, (**m**–**o**) pressure points.

The general structure of the LFIA is reported in Figure 2 and consists of an ensemble of components providing chemical, physical, and mechanical features [35,36,37,38,39,40].

Basically, it appears as a multilayer strip [14]. On a backing plastic support (Figure 2a), a thin layer of porous nitrocellulose membrane (Figure 2b) adheres. The backing support acts as a platform for the assembling of the different components of the test and confers physical rigidity to the device. At the ends, two cellulose or glass fiber pads are pasted—the sample pad and the absorbent pad (Figure 2c,d) [14]. The sample pad absorbs gradually the liquid sample and makes a physical pre-treatment reducing the matrix effects. The absorbent pad works as the driving force for the capillary flow and a sink for the liquid processed through the strip. Between the sample pad and the beginning of the nitrocellulose membrane is stuck another pad, generally made of polyester or glass fiber, called conjugate pad (Figure 2e). On defined regions (generally lines) of the nitrocellulose, solutions containing immunoreagents are dispensed (Figure 2f,g). These can be one or more specific areas called Test lines and one Control line. The role of the Test lines is to give evidence of the interaction with the target molecule(s) and, consequently, the required information. The Control line ensures the correct functioning of the test by binding with the probe independently on the presence of the target. The conjugate pad is impregnated with a suitable labelled immunoreagent solution (usually gold nanoparticles- or latex-labelled antibodies [4,35,37,38,39,40]) and dried (Figure 2h). The assembled strip is enclosed and stored into a plastic cassette (Figure 2i,j) providing a window in the area that includes the reactive regions on the nitrocellulose (reading window, Figure 2k). Another hole is in correspondence of the sample pad, upon which the sample will be introduced (sample well, Figure 2l). The cassette provides some pressure points allowing the contact between the overlapped components, assuring the correct flow of the sample/labelled conjugate mix solution along the whole strip (Figure 2m–o) [39].

**Figure 3:**
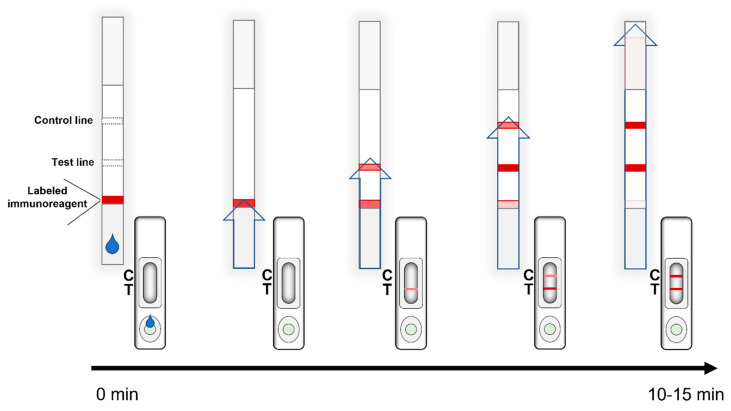
Run of the assay: execution of the test and progression in time. Visually the appearance after the addition of the sample in the sample well changes in intensity until the complete appearance of the reacting bands.

The LFIA starts by applying the liquid sample on the sample pad (Figure 3). The solution resuspends the labelled immunoreagents from the conjugate pad and the analytes and the labelled probe flow by capillary forces along the membrane and through the lines, where immunoreactions take place [14]. Usually, they do not require external reagents for completing the assay besides the liquid sample. Results are quick and easy to interpret, usually without the help of equipment for qualitative assays [4]. In addition, as discussed in Section 7, “Evergreen and new challenges”, the use of reader devices allows to obtain also semiquantitative results, avoiding the subjective results interpretation.

## 3. Literature Review Methodology

The Scopus database was used to search English articles (no review) between 2010 and 2019. The search query consisted of terms considered adequate by the authors to review the literature on the LFIA applications. Because the authors would give to the readers a clear and wide scenario on the LFIA uses, no limitations regarding the field of application were included in the initial research. To simplify the research, the most common nomenclature (lateral flow immunoassay) was searched in title, abstract and keywords, while other nomenclatures referred to the technique were searched just in the article titles. The search query used was (Scopus format): TITLE-ABS-KEY (lateral AND flow AND immunoassay) OR TITLE (lateral AND flow AND assay) OR TITLE (immunochromatographic) AND PUBYEAR > 2009 AND PUBYEAR < 2020 AND (LIMIT-TO (DOCTYPE, “ar”)) AND (LIMIT-TO (LANGUAGE, “English”)). The research has been done in October 2020. Then, the articles were selected following the PRISMA guideline for systematic reviews [41].

**Figure 4:**
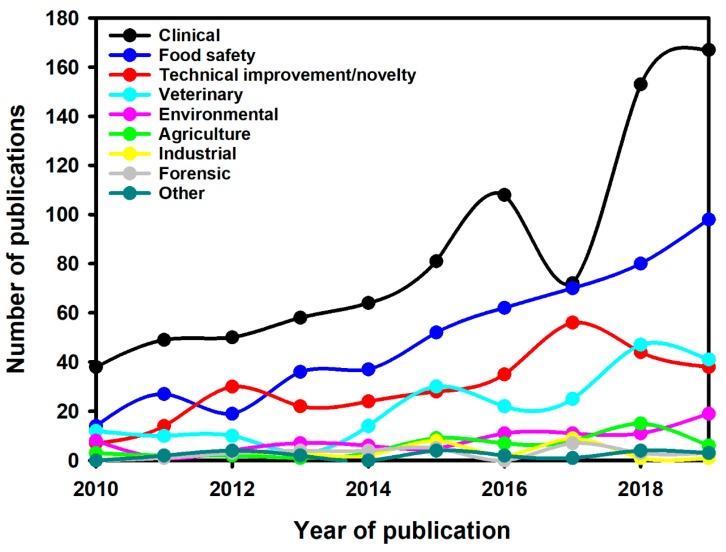
Number of publications per year for different classes of LFIA application from 2010 to 2019.

A total of 2165 document results were obtained and screened to assess their inclusion in the present review. Therefore, 2165 articles title and abstract were examined to remove publications that were not relevant. Eighty-seven articles were excluded because they were not related to the LFIA topic; thus, 2078 articles were identified as relevant. Subsequently, the articles were initially classified in the following classes: clinical, food safety, technical improvement/novelty, veterinary, environmental, agriculture, industrial, forensic, and other. While most of these classes are self-explained, we want to point out the meaning of some of them. Publications in which the application was just a proof-of-concept or a model system to demonstrate advances in the technique were classified as “technical improvement/novelty”. Publications regarding plant diseases were classified as “agriculture”. Publications regarding the quality control of industrial processes were classified as “industrial”. The output of this classification process is reported in Figure 4.

## 4. Applications

### 4.1. Clinical Applications

Currently, clinical diagnostic tests often rely on analyses performed in a central laboratory giving results after several hours or even days [4,8,42]. Considering that in many cases a timely decision can drastically affect the clinical outcome, the potential benefits of LFIA use in the clinical field are self-evident [4,8,42]. LFIA can make a valuable contribution in screening, help in diagnosis, prognosis, monitoring, and surveillance [4]. The immediate clinical assessment may have a tremendous impact in the disease management reducing the workload, enhancing the workflow, improving clinical care and patients’ outcomes, and potentially decreasing costs [4,42]. The use of LFIAs can allow patients to receive the diagnosis and the specific treatment during the same consultation, reducing the number of clinical visits avoiding referral and problems related to delay in starting the therapy [42]. Moreover, long-term benefits should not be underestimated. For example, LFIA can help distinguishing between bacterial and viral infections, thus identifying cases where antibiotics therapy is needed, limiting the misuse of these drugs that lead to accelerate the antibiotic resistance.

LFIAs have the inherently suitability for use outside the laboratory setting [42]. Therefore, in addition to those performed by healthcare professionals in hospital laboratories, LFIAs are used in hospital wards, clinics, health centers, physicians’ offices, and even patients’ home in the self-testing format [4,36,42]. It is undeniable that the most emblematic test in the clinical field is the pregnancy test [7,36]. However, many others LFIAs have been developed and used over time for different clinical purposes [4].

The great success of LFIAs in the clinical field can be associated with the direct impact on the human health and also because the very first applications have had clinical purposes. Moreover, it is worth noting that, even considering the relative complexity of biological fluids, they are quite limited in number. For example, the most used biological matrices are venous or capillary blood, saliva, urine, nasopharyngeal swabs, and stools [4,18,40]. Therefore, once defined, a suitable sample treatment the analysis becomes quite easy. Sample handling and/or treatment can include multiple variations depending on the sample type, including plasma separation from fingerstick or venous whole blood, filtration, cellular lysis for bacteria or viral intracellular antigens, and breakup of mucins in saliva or respiratory samples or changes in pH of urine, to name a few examples [43]. In some cases, these kinds of treatments can be performed using particular sample pads, therefore without additional steps [4]. Moreover, the recognition elements usually employed in LFIA (antibodies) are inherently inclined to perform very well in biological fluids.

In the considered 10-year period, the detection of infectious diseases accounted for 69% of the total applications (50% bacterial, 36% viral, and 14% caused by other organisms), followed by the detection of endogenous markers and biomarkers (28%), while the remaining 3% mainly comprises the detection of drugs and their monitoring. It is not surprising that the vast majority of applications are for infectious diseases since the rapid and early identification of an infected person allows limiting the infection spread itself and increases the probability of patients’ recovery. Other driving forces that boosted the application in the infectious diseases field are the willingness and the efforts of the World Health Organization (WHO) to face the so-called priority/key diseases. In fact, this trend also reflects the LFIAs reported in the list of essential in vitro diagnostic (EDL) issued in 2018, and last-updated in 2019, by the WHO. The EDL outlines a group of IVDs that are recommended by WHO for use at various levels of a tiered national health care system with the aim of providing evidence-based guidance and serving as reference to Members States who are developing and/or updating lists of national essential IVDs for defining universal health coverage interventions, as well as selecting and implementing such IVDs [44]. The EDL mainly comprises the detection of infectious diseases like cholera, cryptococcal meningitis (in people with advanced HIV disease), dengue virus, hepatitis B and C, HIV, influenza A and B, malaria, syphilis, tuberculosis, and visceral leishmaniasis [45]. In addition to these LFIAs application, in the EDL are also listed the pregnancy testing, and the detection of C-reactive protein and procalcitonin. Considering that the EDL will be expanded and updated annually promoting progress towards the goal of universal health coverage, it can be expected that several new applications will be added in the near future.

### 4.2. Food Safety Applications

The production and the commercialization of food that does not cause a risk to the consumer is a major global concern because it is known that unsafe food can cause over 200 diseases that can range from digestive tract infection to cancer. Moreover, every year 420,000 people die and more than 600 million fall ill from eating contaminated food [46]. To reduce these frightful numbers, the food safety must be guaranteed along the whole food chain in order to avoid the presence of potentially toxic substances such as veterinary drugs, heavy metals, pesticides, toxins, fertilizers, pathogens and undeclared allergenic ingredients [1,13,47]. Following good agricultural and good manufacturing practices is the cornerstone to pursue this goal [48]. However, only a widespread network of controls can further minimize the risk [2,47,49].

The use of LFIAs in food safety can help the management of foodborne risks by increasing the number of analyses, making them accessible, fast, and inexpensive, allowing to monitor food safety alongside production chain, from raw materials to ready-to-eat products. Moreover, their simple and fast use makes them the ideal device to be implemented in the Hazard Analysis and Critical Control Points (HACCP) procedures [49].

Compared to LFIAs devoted to the clinical diagnostic field that have to deal with limited sample matrices, LFIAs for food safety have to tackle an additional challenge due to food matrices that can be as diverse as complex and numerous [40,47]. To have an idea of such a diversity, we can just think of how it can be different to analyse cereals, biscuits, meat, spices, milk, alcoholic and non-alcoholic beverages, drinking water, etc. Accordingly, the needs of every new device become very different. For example, monitoring of mycotoxins in food and feed may requires very different approaches along the entire production chain, and in all these scenarios, the LFIA should be robust and adaptable enough to ensure a valid analysis [47,50]. Based on the properties of the target analyte and the related matrix, sometimes it can be necessary to use an organic solvent to perform the extraction. However, antibodies and LFIA components (especially the nitrocellulose membrane) have a restricted tolerance for organic solvents. Therefore, a challenging task in assay development may be determining an optimal solvent system for both analyte solubility and method performance. Sometimes an additional dilution step in a proper buffer is essential to provide a suitable medium to allow more efficient analyte detection.

In the considered 10-year period, the most frequent application in this field was the detection of toxins (39%), followed by drugs (30%, mainly antibiotics), and pathogens (22%), while pesticides, adulterants, and allergens accounted for the remaining 9%.

### 4.3. Veterinary Applications

Veterinary medicine mainly applies to companion animals like dogs, cats, etc., and to livestock animals, i.e., animals that are considered as an asset (cows, sheep, poultry, pigs, etc.). Veterinary services are essential to assure animal health and, in a broader context, to prevent and control animal diseases, including those transmissible to humans (zoonoses), to ensure the sanitary safety of world trade in terrestrial and aquatic animals and animal products, and to improve animal welfare worldwide [51]. Veterinary medicine shares several similarities with clinical diagnostic for human health. Even in this field, a timely right decision can drastically affect the outcome.

Effective surveillance, early detection, transparency, and rapid response mechanisms in the event of disease outbreaks are the key to prevent and control animal diseases. Controlling the diseases that affect terrestrial and aquatic animals and improving the welfare of these animals are the core of the World Organisation for Animal Health (OIE, historic acronym of the Office International des Epizooties) mandate [52], not only because it has obvious health benefits for both animals and humans, but also considering the impact on the sustainability of socio-economic and ecological systems.

Nowadays, animal diseases can spread even more because of the exponential growth in trade and tourism. The official sanitary status of countries regarding animal diseases has become a key factor to preserve animal health, public health, and a safe international trade. The 182 OIE member countries undertake to report the terrestrial and aquatic animal health situation in their territory in a timely and transparent manner. The disease identification and the outbreak report must be as fast as possible to minimize their negative impacts. Of course, the most worrisome diseases are the infectious ones due to the easiness of spread and to their dramatic consequences (large-scale culling of livestock in some cases). In this context, the use of rapid screening tests may help and accelerate the infectious disease identification and thus direct immediate and focused actions to stem its spread. Moreover, considering that 60% of the pathogens that affect humans are of animal origin [51,53], it is easy to understand the crucial role of the early detection of these diseases at their source in animals.

The use of diagnostic rapid tests in the veterinary field has increased in the last decades due to the willingness of pet owners to keep their pets healthy, and to the increased acceptance from farmers of the benefits of near-animal testing [54]. The ability to provide an immediate answer leads to better management and intervention strategies. Currently, veterinarians use rapid tests to screen commercial livestock and household pets for several medical conditions. These tests have potential utility in many veterinary settings, including private clinics, academic veterinary medical centres, the community in remote area, and for research applications in academia, government, and industry [55].

Another driving force for the increasing acceptance of diagnostic rapid tests in veterinary medicine is the increasing concern of customers about antibiotics and transmissible diseases in milk, eggs and meat, and to the widespread public concern over the spread of diseases through populations of animals [54]. Some of these concerns are strictly related to food safety and human health and they are added to the global concern regarding antimicrobial resistance that causes food production losses, poor animal welfare and extra costs.

In many countries, providing antimicrobials in the feed and water of farmed animals to prevent disease or to stop its spread accounts for a greater proportion of total antimicrobial use in farming than the treatment of sick animals [56]. Such practices often occur without precise diagnosis and without confirmed disease presence.

In the World Health Assembly held in May 2015, it appeared clear that the antimicrobial resistance crisis needed to be managed with the utmost urgency. Consequently, in the same year, the WHO launched a global action plan on antimicrobial resistance, which outlines five strategic objectives [57]. Among these objectives, the focus was on optimizing the use of antimicrobial medicines in human and animal health and on increasing the investment in diagnostic tools. It was pointed out that effective, rapid, low-cost diagnostic tools were needed for guiding optimal use of antibiotics in human and animal medicine, and that such tools should be integrated into clinical, pharmacy and veterinary practices. Ensuring that rapid and affordable point-of-care tests are available for critical animal diseases where antimicrobials are most commonly used will reduce the misuse of these drugs in animal treatment improving antimicrobial stewardship and animal health and welfare [58]. On this basis, reliable LFIAs can help veterinarians and farmers to make a responsible and prudent use of antimicrobial agents, thus maintaining their therapeutic efficacy.

Considering the huge variety of animal species and their related peculiarities, LFIAs for veterinary use have to deal with a multitude of matrices like serum, urine, buccal and nasal secretions, mammary secretions, milk, faeces, respiratory exhalations, etc.

As imaginable, in the considered 10-years period, the detection of infectious diseases accounted for 93% of the total LFIA applications (51% viral, 27% bacterial, and 22% caused by other organisms), followed by the detection of drugs.

### 4.4. Environmental Applications

The environmental pollution has become a global crucial issue. Several pollutants and contaminants enter the environment either because of anthropogenic activities like industry, agriculture, transport, everyday life, etc., or through naturally occurring event [59,60]. Pollutants and contaminants can be air-, soil-, or waterborne, and may move from one medium to other (for example, soil to water). They can directly and indirectly affect human health, and the socio-economic development of a country [61]. Therefore, a major concern lies in detecting and monitoring air, soil, and water pollutants [62].

The environmental monitoring is the essential and regulated activity that aims at safeguarding the environment and protecting living beings from exposure to toxic pollutants, contamination sources, and pathogens. This monitoring can be divided in three macrophases: (i) initial monitoring that is required to identify the levels and effects of certain pollutants on the environmental media; (ii) identification of the sources of these pollutants in order to effectively inform the policymakers; (iii) continued monitoring of environmental conditions that is important to verify if environmental quality standards are fulfilled (verify for concentrations in water, sediment and biota that must not be exceeded) and to assess the usefulness of the regulatory actions, once implemented [63].

The pollutants monitoring allows identifying the spatial distribution of contaminants to determine which sites are at risk and examine temporal trends at different sites to determine if the situation is improving or worsening [64]. This process generally provides data on average concentrations in environmental media, while peak concentrations are obtained when the measurement is performed at the waste point.

The control of contaminant levels in the environment is costly, often time-consuming, and labour-intensive, especially considering the largeness of the environmental media. The accomplishment of environmental analyses requires a great deal of advanced analytical chemistry expertise, together with complex and expensive instrumentations [60]. In fact, chromatographic and spectroscopic methods are used in the laboratory for detection of pollutants, and polymerase chain reaction (PCR) based detection is usually used for identification of pathogens [62]. Therefore, alternative approaches that can provide on-site, high-throughput, easy, and real-time testing in a speedy manner are highly demanded to perform a cost-effective monitoring [65].

LFIAs can be used as environmental quality monitoring tools in the assessment of inorganic and organic pollutants, and biological contaminants [40]. While this kind of sensors are not usually used for monitoring air quality, they are mainly used for monitoring water- and soilborne contaminants [40,62]. In this context, contaminants detection in soil involves extraction procedures, while water samples usually need minimal pre-treatment. Nevertheless, improvement in the analytical performances (mainly the sensitivity) are a big challenge for the use of LFIAs because the permissible levels set by regulatory agencies can be very low for some substances [66].

In the considered 10-year period, the detection of heavy metals has been the prevalent application (37%), followed by pesticides (14%), algae (11%), pathogens (10%), toxins (8%), drugs (6%) and other compounds (14%).

Unlike the previous application fields in which one target class (or two for food safety) accounts for ca. 70% of the total applications (or even ca. 90% for veterinary), target classes in environmental application are more assorted. This can be explained considering that, historically, several substances have been worried about the ecosystem. In fact, heavy metals, pesticides, pathogens, and toxins—that all together account for ca. 70% of total applications—have been considered as some of the most worrisome environmental pollutants, led by heavy metals mainly due to industrial wastewater [67].

Moreover, in the last few years, the concept of emerging contaminants (ECs) has become more and more pressing, and consequently more and more substance classes needed to be monitored to safeguard the environment. ECs include a wide range of chemicals, such as persistent organic pollutants, pharmaceuticals, personal care products, endocrine disrupting compounds, sweeteners, nanoparticles, etc. [68,69]. Among ECs, antimicrobials have become an increasing concern, due to the increasing likelihood that microbials develop resistance against drugs and can accumulate in wildlife. As stated in previous sections, antimicrobials resistance is a global concern; in this context, the One Health action plan was launched, in 2017, by the European Commission as part of the WHO’s global One Health program that recognizes the interconnectedness of human health, animal health, and the environment for sources of resistant bacteria [70]. Monitoring the environment for antimicrobial resistant species is also crucial because it might help to predict clinically relevant infection outbreaks [71].

### 4.5. Other Applications

The previous applications accounted for more than 90% of LFIA total applications. Nevertheless, LFIA is also applied in other fields. In the considered 10-years period, the remaining applications are dominated by the use in the agriculture field (40%) where LFIAs mainly help in the detection of plant diseases, followed by the quality monitoring of industrial products and/or processes (22%) and by applications in the forensic field (22%) like detection of blood and illegal substances. Finally, niche applications have been also reported in the literature from cultural heritage to biotechnology [72,73]. Among them, the detection of biohazard compounds is extremely relevant. In fact, some of the compounds mentioned in the previous sections like bacteria, viruses, fungi, and toxins that threat the human health may be used in biowarfare attack; therefore, their prompt detection can make the difference in the effectively contrast of bioterrorism.

## 5. Multiplex LFIAs

The simultaneous analysis of more than one analyte in a single test, i.e., the multiplex detection, is increasing its relevance in several fields. The capability of multiplexing can significantly improve the efficiency of testing and reduce costs while enhancing high-throughput detection. It is strongly requested for those applications in which advanced decision-making is needed or availability of samples is limited [32].

Multiplex testing has become more and more requested in contemporary clinical diagnoses [12]. With the increasing numbers of (bio)markers discovered, there is often a need to detect several (bio)markers simultaneously to generate meaningful or conclusive information, especially when a single (bio)marker may be indicative of more than one cause or when a particular condition is influenced by more than one parameter. In addition to this, due to the increased number of compounds to be monitored, the possibility to obtain information regarding all the involved targets within the same single test has become highly demanded in food safety, veterinary, and environmental monitoring as well.

The simultaneous detection of multiple analytes is mainly realized using the design of several Test lines in a single strip allowing the targets discrimination through the spatial resolution [30,32,74,75,76]. Combining multiple lines is the most direct way of increasing the detection capability while retaining the merits of single LFIA systems. This approach has been mostly used in the detection of food borne bacterial pathogens and mycotoxins but has also been described for the detection of single nucleotide polymorphisms, parasites, and antibodies [30]. The evaluation of a test with several Test lines may be not so user-friendly; therefore, integrated reader system can represent a useful, but more expensive solution, reducing their potential use in low-resource environment. However, misleading interpretation can be avoided using multicolour labels, simplifying the visual interpretation without compromising the cost-effectiveness of the test.

In addition to the spatial resolution strategy, the separation of reaction sites using single strips for each specific target, arranged in a multichannel structure, has also been exploited [77,78,79]. In this alternative strategy, no risk of reciprocal interference exists between assays. However, the sample volume required increases with the increase of strips number arrayed together, as well as the fabrication costs and reagents consumption.

Recently, the use of a single strip with a single Test line has also been applied for multiplexed detection of target molecules, exploiting surface-enhanced Raman scattering [80,81] and colorimetric detection [82,83]. Moreover, most recently, Cavalera et al. proposed a two-parameter multiplexing LFIA strategy (x2LFIA), which combines the spatial resolution with colour encoding approach to expand the number of information achievable within a single strip test, obtaining tetravalent information in a two-line and two-colour assay [84].

The trends for the multiplex LFIAs, in the considered 10-year period, are reported in the following section.

## 6. LFIA Applications Trends

Interestingly, in the 10-year period, the food safety application showed the highest growth with a CAGR of 21%, while clinical application grew at a CAGR of 16%, followed by veterinary (13%) and environmental (9%). Clinical application most likely will reach and overcome again the growth of food application in 2020–2021 due to the COVID-19 pandemic and the related LFIA developed to detect the viral antigens or the antibodies against them. In the same period, the efforts of researchers to enhance the LFIA technique can be highlighted from the 18% CAGR of articles publication regarding technical improvements.

**Figure 5:**
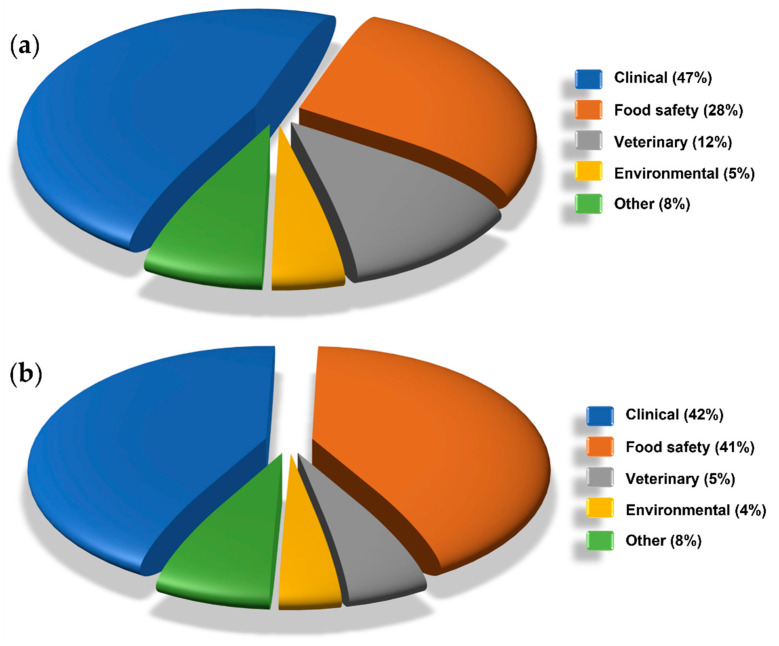
Overview of (**a**) LFIAs and (**b**) multiplex LFIAs application fields in the period from 2010 to 2019.

Excluding the articles mainly focused on the technical improvements (298) and unifying in the category “other” the applications related to agriculture, industrial, forensic and other (e.g., biotechnology, cultural heritage, etc.), the breakdown by LFIA application is reported in Figure 5a. As expected, in the considered 10-year period, the clinical application accounted for the majority of the total LFIA applications (almost half), followed by food safety, veterinary and environmental.

Another interesting trend can be drawn for the applications in which two or more target analytes are detected simultaneously (multiplex analysis). The multiplex analysis grew at a CAGR of 26% and the breakdown by field of application is reported in Figure 5b. Clinical and food safety applications share equally almost the totality of the multiplex segment. This breakdown can be explained remembering the essential role of multianalyte detection in clinical diagnostic and considering all the advantages derived from detecting in a single analysis all the compounds to be monitored in the same food sample.

The great significance that the scientific community is giving to the multiplex analysis can be evaluated also by monitoring the impressive CAGR in clinical (37%) and food safety (57%) fields.

In order to provide an overview of the most recent and hot applications, in Table 1, Table 2, Table 3, Table 4 and Table 5 we also reported some of the most interesting examples of LFIA for each relevant field mainly covering the 2020–2021 time frame.

## 7. Evergreen and New Challenges

Although LFIAs are inherently the ideal qualitative screening method, they have been asked to be a quantitative method that allows the analyte ultrasensitive detection. A lot of efforts have been made in this direction thanks to the use of new labels and/or strategies to improve the sensitivity, and to the use of dedicated strip readers [15,16,17,34,267].

**Figure 6:**
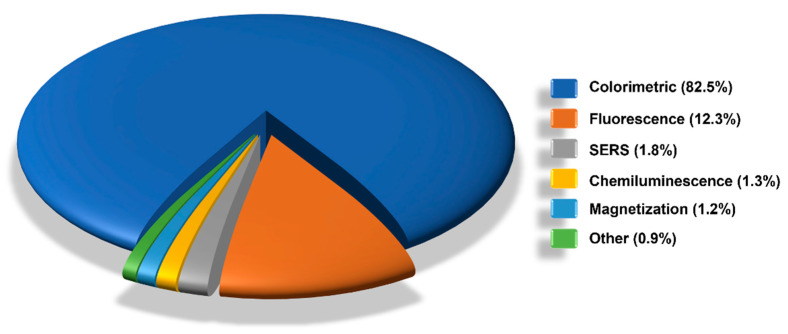
Overview of LFIA detection methods in the period from 2010 to 2019.

The employment of more or less sophisticated external readers allowed the quantitative analysis of LFIA strips by measuring the intensities of the signals generated at the reactive lines. While on the one hand the use of readers increases the cost per analysis, on the other it allows data digitalization, tracking, storage, and transmission reducing interpretation and transcription errors, thus ensuring testing quality and control [18]. In this sense, we are also witnessing an increasing exploitation of the built-in smartphone technology to be used as LFIA reader, as increasingly reported in the literature [122,132,267,268,269,270,271,272,273,274,275]. Moreover, the use of strip readers paved the way for the use of alternative detection methods (fluorescence, chemiluminescence, etc.) allowing better performances and quantitative measurements. In fact, the colorimetric detection has dropped from 93.7% in 2010 to 78.1% in 2019 mainly in favour of the fluorescence (from 3.8% to 15.8%) and surface-enhanced Raman spectroscopy (SERS) detection (from 0% to 3.6%). However, as can be observed from the breakdown by detection methods reported in Figure 6, in the period from 2010 to 2019 the colorimetric detection still dominates (82.5%), followed by fluorescence (12.3%) and SERS (1.8%) detection.

**Figure 7:**
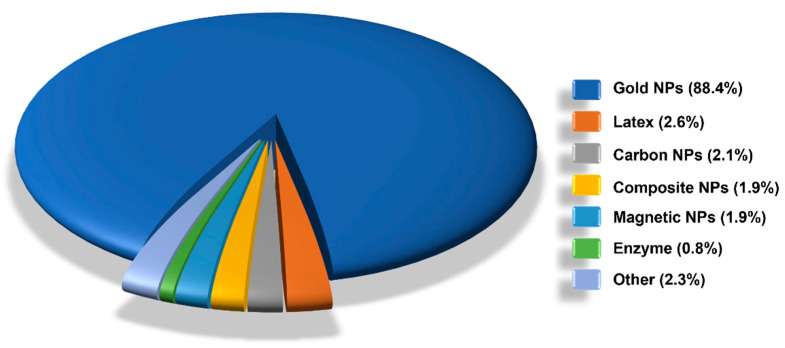
Overview of LFIA labels used in colorimetric detection in the period from 2010 to 2019.

As additional information, analyzing the breakdown by LFIA colorimetric labels employed in the considered period (Figure 7), the gold nanoparticles accounted for the 88.4% followed by latex (2.6%) and carbon nanoparticles (2.1%). Most recently, the use of gold nanoparticles has slightly decreased (from 89% in 2010, to 81% in 2019) mainly in favor of the use of composite nanoparticles that has grown from 0% to 6%.

Despite the several advantages resulting from the use of LFIAs, there are still some concerns regarding the routine use of these devices. For example, some veterinarians expressed discontent about the increasing use of LFIAs by farmers themselves without veterinary supervision [56]. These concerns are mainly due to the risks associated with such role diversification (somehow also related to the professional authority delegitimization when tests are being used directly by the end-user) and diagnostic simplification including samples contamination, poor control of the environment, misinterpretation of the results and, as a potential consequence, unnecessary or inappropriate drugs use. However, the concerns regarding the correct use of the device can be addressed through (i) unambiguous instructions sheet, (ii) training of the end-user, (iii) limiting the number of steps to be performed, (iv) meticulous design development, and (v) performing accurate test usability. On the other hand, as previously mentioned, the possible misinterpretation of the results can be solved using reader devices.

Another great concern regards the reliability of tests, their diagnostic power and accuracy compared with laboratory test procedures, concern that is shared also by some general practitioners that consider this aspect, together with clinical management, as the most important aspect for implementation in routine use [276]. Reliable qualitative and/or quantitative results can only be achieved by means of a deep validation of the LFIA device and meticulous quality control. This aspect has been underestimated in most publications regarding the development and application of new LFIAs. While one might think that this aspect should only concern manufacturers, it is undeniable that the researchers’ community can be a fundamental and ground-breaking support to draw up or to test validation protocols as shown by the interesting and pioneering studies of Lattanzio et al. [277,278,279,280,281] and by a few other researchers [282,283]. In this regard, very recently, Bheemavarapu et al. proposed a promising tool for the quality assessment of LFIA strips batches and to assure a robust validation of the test itself through an image processing-based algorithms to evaluate sample flow abnormalities or membrane irregularities [284].

An increasing use of LFIAs to obtain quantitative and reliable results will only be possible if the highest standards of quality and performance will be met. In this regard, the effective implementation of LFIA, in routine use, can be boosted through national and international regulations that have the aim of guaranteeing the safety, quality, and effectiveness of in vitro diagnostic tests.

Nevertheless, sometimes the impact of new regulations can have a negative effect on the use of diagnostic tests, especially at the beginning of their implementation. For example, in 1988, there was the introduction of the Clinical Laboratory Improvement Amendments (CLIA) regulations that include federal standards applicable to all U.S. facilities or sites that test human specimens for health assessment or to diagnose, prevent, or treat disease. A few years after the CLIA regulations entered into force, it has been reported that more than 64% of physicians cited CLIA 1988 as a factor in their decision to reduce or eliminate in-office testing. The most striking effect of CLIA 1988 appeared to be on pediatric practices and practices in rural areas, of which more than 70% have reduced or eliminated onsite testing, thus sending patients and specimens to outside facilities, compromising patient’s access to timely quality testing, resulting in delays in diagnosis and treatment [285]. However, after an initial negative effect, the in-office testing thrived again driven by the physicians’ belief in the POCT utility, by the economic interest and by the fact that hundreds of tests have been approved for the waived category.

Moving to the present day, a radical improvement is expected to be obtained in the next years thanks to the new European Regulation on in vitro diagnostic medical devices that establishes more restrictive requirements for IVDs quality, safety, and reliability [286] whereby most of the self-certified IVDs will have to be re-certified through the conformity assessment by a notified body. Therefore, clinicians and end users will no longer have to rely only on the good faith of the manufacturers, but they will also have the assurance that the device has been approved by a third party. Of course, the same care should be applied also to the other fields of applications.

## 8. Future Perspectives

We have already outlined the importance of multiplex LFIAs in several application fields. The number of multiplexing LFIAs is expected to grow exponentially because they improve the efficiency of testing and because more and more diagnostic questions require the detection of various analytes to explain a particular condition. However, the multiplexing strategies applied up to now have inherent limitations regarding the maximum number of analytes to be detected simultaneously. A possible solution might be the use of the microarray format that owns the right peculiarities to improve the multiplex capability. Although very promising, this approach has been reported just a few times in the literature [2,287,288,289,290] and the reason of this under-use may be mainly associated to the possible microfluidic modification in using a microarray pattern on the membrane instead of the lines pattern, and to the more complex readability of the results that may cause misleading results interpretation. A deeper and more accurate study of the microarray LFIA and the use of a reading device could help its spread in the near future.

It has already been outlined that LFIAs own all the features to satisfy the ASSURED criteria. Recently, these criteria have been revised by adding three criteria, namely, real-time connectivity, ease of specimen collection, and environmental friendliness to assemble the so-called REASSURED criteria [239]. Unsurprisingly LFIA can already meet almost all of these additional desiderata.

Regarding the real-time connectivity, the use of smartphones as strips reader (as previously mentioned) or hand-held readers equipped with a connectivity module allow to deal with this task in an easy way.

The specimen collection is usually very simple; moreover, the following sample preparation does not require multiple steps, apart from cases where a dilution or extraction must be done. However, in most cases, solutions transfer and manual mixing are enough to prepare the sample for the analysis. Because specimen collection and treatment are the basis to perform an accurate analysis, the easiest standardized protocol should be provided to the end user in order to minimize operations and user errors. In this regard, innovations and improvements are still highly required in order to provide cartridges for sample preparation that could be integrated or connected directly to the strip.

In comparison to other analytical techniques, LFIA can be considered as a green and environmentally friendly technique both because most of the devices do not use organic solvent (except for the extraction of some analytes from solid sample) and because the sample can be analysed minimizing ancillary costs, for example minimizing the energy consumption, avoiding sample transportation and the need for the cold chain, etc. However, being a disposable device, the use of plastic cassettes is an issue for the environment and therefore the use of recycled plastic is highly desirable. The use of biodegradable components could also be desirable, even if most of the LFIAs for professional use (in the clinical field) must be incinerated due to possible biohazard. Notwithstanding the exceptional properties of the nitrocellulose as solid support for the LFIA platform, researchers [291,292,293] are trying to replace its use with the cellulose that, in addition to further reducing costs, would have a reduced impact on the environment because its production process requires less chemicals in comparison to the nitrocellulose production. Most recently, promising results have been obtained by Adrian Elter et al. that developed cellulose-based LFIAs [294]. However, their strategy consists in the use of carbohydrate binding module-fused antibodies and therefore it cannot be directly applied using currently available antibodies without additional steps.

The unprecedented pressure on laboratories and health care systems, due to the COVID-19 pandemic, caused the postponement of almost all medical examinations, screening, and diagnostic tests not related to the COVID-19 spread. As a consequence, the need of decentralized rapid diagnostic tests has been highlighted now more than ever because they can be regarded as useful allies to help in diagnosis without increasing the workload. However, they also have to deal with the recent discontent originated from their use to detect antibodies against SARS-CoV-2 or to detect the virus itself, and with the negative publicity received from media and people’s opinion. This situation was mainly due because (i) the serological detection of antibodies against SARS-CoV-2 was used to diagnose the infection and to perform retrospective epidemiological analysis, and (ii) the first antigenic LFIAs had poor sensitivity, resulting unreliable [295,296,297,298,299].

Although the recent outbreak may be a great opportunity for the durable implementation of LFIAs to loosen the testing pressure on health care facilities, it has also highlighted how the effective use of rapid tests may occur only guaranteeing their reliability (certifying, though a deep validation, the test accuracy, precision, sensitivity, specificity, etc.), and their meditated and cautious use. The ease of use must not compromise analytical performances, otherwise all the benefits deriving from the use of the LFIAs would be lost, especially if they are used for the wrong purpose.

## 9. Conclusions

After about half a century from its first development, the LFIA technique is increasingly used thanks to its global scalability and to its features that allow reducing costs and workload, while enhancing the workflow efficiency improving the turnaround time. LFIAs have become one of the reference point-of-need tests to obtain results in a timely manner without the need of high-sophisticated and high-cost laboratory equipment, in clinical, food safety, veterinary, and environmental testing.

The interest of the scientific community for the LFIA is still very high, as can be noted by the increasing number of research papers aiming to improve one or more aspects of the technique. The sensitivity enhancement and the quantitative output have been the most studied features during the last decades. Nevertheless, the colorimetric detection is still the most widely used detection method, and gold nanoparticles still dominate the colorimetric labels scenario.

Most recently, the environmental friendliness has become a leading topic also stimulated by the introduction of the REASSURED criteria, as a revised and extended version of the ASSURED criteria coined to describe the ideal POCT to be used in the developing world, and then became the gold standard features for any point-of-need test. Likewise, the easy specimen collection and treatment should dictate the next advances and improvements in LFIA.

In the period from 2010 to 2019, multiplex LFIAs showed the highest CAGR and are expected to continue to grow due to their unquestionable benefits. The main field of application is confirmed as the clinical one. However, it is worth noting that, in the same period, the number of publications regarding the LFIA use related to the food safety field has grown with a CAGR higher than the clinical field. However, this trend is expected to be overturned in 2020–2021 considering the plenty of publications about the use of LFIA related to SARS-CoV-2.

The global outbreak of SARS-CoV-2 itself may be a boost to the LFIAs routine use both in poorly supplied structures in order to allow the decentralization of primary care and the simplification of the diagnostic process, and even in highly advanced and organized facilities that own sophisticated instrumentations. At the same time, an increasing trust in the LFIAs use in the clinical field will continue to foster more and more their use also in other fields.

However, a massive use is only possible guaranteeing the reliability of the LFIA devices and of any kind of advancement related to the device itself. In this regard, an accurate validation is essential to obtain reliable rapid tests that can be really helpful, establishing more and more as the right tool to answer, in timely manner, the right question.

## Figures and Tables

**Table 1:** Overview of the most recent and hot LFIA applications in the clinical field.

Application Field	Target		Matrix	Reference
Clinical	Viruses and related infections	Ebola virus	Whole blood, plasma	[85,86,87]
		HIV-1 and -2	Blood, serum	[84,88]
		Noroviruses	Stool	[89]
		Influenza A/B	Nasopharyngeal (nasal) swab	[90]
		Chikungunya virus	Serum	[91]
		Dengue virus	Blood	[92]
		Herpes simplex virus type 2	Plasma, serum	[93]
		SARS-CoV-2	Serum, blood, saliva	[94,95,96,97,98,99,100]
	Bacteria and related infections	Brucellosis	Serum, plasma, whole blood	[101]
		Helicobacter pylori	Stool	[102]
		Pneumococcal pneumonia	Pleural fluid	[103]
		Plasmodium falciparum infections	Whole blood	[104]
		Scrub typhus	Serum	[105]
	Other infectious diseases	Burkholderia pseudomallei infections	Blood, urine, other bodily fluids	[106]
		Pneumocystis pneumonia	Serum	[107]
		Strongyloidiasis	Serum	[108,109]
		Candidiasis	Pharyngeal swabs	[110]
		Progressive disseminated histoplasmosis	Serum	[111]
		Toxoplasmosis	Serum	[112]
		Allergic bronchopulmonary aspergillosis	Serum	[113,114]
		Fascioliasis	Serum	[115]
		Chagas disease	Serum	[116]
		Cerebral angiostrongyliasis	Serum	[117]
	Other diseases	Systemic lupus erythematosus	Serum	[118]
		Sepsis	Serum	[119]
		Acute hyperglycemia and diabetes mellitus	Serum	[120,121]
		Diabetic retinopathy	Urine	[122]
		Alpha thalassaemia	Whole blood	[123]
		Kidney injury	Urine	[124]
	Cancers	Gastric	Plasma	[125]
		Cervical	Urine	[126]
		Ovarian	Serum	[127]
		Bladder	Urine	[128]
		Prostate	Urine	[129]
	Health status (bio)markers	Folate	Serum	[130]
		Hormones	Saliva, urine, serum	[131,132,133,134,135,136]
		Cardiac biomarker	Serum, finger-prick blood	[137,138,139]
		Ferritin	Serum	[140]
		Myoglobin		
	Therapeutic drug monitoring	Tenofovir	Urine	[141,142]

**Table 2:** Overview of the most recent and hot LFIA applications in the food safety field.

Application Field	Target		Matrix	Reference
Food safety	Toxins	Botulinum neurotoxin type A and Staphylococcal enterotoxin B	Milk, grape juice	[143]
		Staphylococcal enterotoxin B	Milk, honey	[144]
		Tetrodotoxin	Crucian, clam	[145]
		Amatoxins	Mushrooms	[146]
	Mycotoxins	Aflatoxin B1 and fumonisins	Maize flour	[82]
		Zearalenone	Maize, cereals	[147,148]
		T-2 toxin	Tap water	[149]
		Aflatoxin B_1_, zearalenone and deoxynivalenol	Feedstuff	[150]
		Fumonisin B1 and deoxynivalenol	Grain	[151]
	Antimicrobials	Tylosin and tilmicosin	Milk, pork	[152]
		Sulfamethazine	Egg, honey, pork	[153]
		Bacitracin	Milk	[154]
		Diclazuril	Chicken	[155]
		Lincomycin	Milk, eggs, honey	[156]
		Carbadox and Cyadox	Chicken breast	[157]
		Bacitracin zinc	Milk	[158]
		Lincomycin and tylosin	Milk, eggs, honey	[159]
		β-lactams	Milk	[160]
		Imidocarb	Milk, beef	[161]
		Colistin and bacitracin	Milk	[162]
	Bacteria	Staphylococcus aureus	Orange juice, lettuce salad, fish	[163]
		Escherichia coli O157:H7	Milk, beef, pork, chicken, bread, jelly	[164,165]
		Salmonella spp.	Chicken, eggs	[166,167]
		Campylobacter jejuni	Milk, chicken	[168]
		Vibrio parahaemolyticus	Clam, white clam, flower clam, razor lam, yellow croaker, fresh shrimp	[169]
	Allergens	Milk casein, egg chicken albumin, hazelnut protein	Bakery products	[170]
		Tropomyosin	Various food products	[171]
		Casein and β-lactoglobulin	Several food matrices	[172]
		Major peanut allergen	Peanut oils	[173]
		Parvalbumin	Fish	[174]
		Gluten	Grain flours, food dough, burger patty, ice cream, soup	[175,176]
		β-conglycinin	Skimmed milk	[177]
	Hormones	Dexamethasone	Milk, pork meat	[178]
		17β-estradiol	Chicken, fish, prawn, pork	[179]
		Diethylstilbestrol and estradiol	Milk, shrimp tissue	[105]
	Pesticides	Triazophos	Cucumber	[180]
		Spirotetramat and spirotetramat-enol	Wine, grape juice, and grapes.	[181]
	Adulterants/food identification/illegal additives	Melamine	Milk, animal feed;	[182,183]
		Specific buffalo’s milk protein	Cow’s milk	[184]
		Saffron genomic DNA	Dried herbal materials	[185]
		Duck meat	Beef meat	[186]
		Horse and donkey meat	Several foods	[187]
		Horse, pork beef, sheep meat	Fresh meat	[188]
		Pork meat	Several meats	[189]
		Goose meat	Raw, cooked food products	[190]
		Chicken meat	Meat products	[191]
		Horse meat	Raw, processed meat products	[192]
		Sibutramine	Diet food	[193]
		Chlorpheniramine	Herbal teas	[194]

**Table 3:** Overview of the most recent and hot LFIA applications in the veterinary field.

Application Field	Target		Matrix	Reference
Veterinary	Viruses and related infections	African swine fever	Blood, spleen, tissue	[195,196]
		Rabies	Brain tissue	[197,198]
		Porcine epidemic diarrhea	Colostrum, stool	[199,200]
		Bovine rotavirus	Stool	[201]
		Avian leukosis virus	Chicken meconium	[202]
		Avian infectious bronchitis virus	Chicken throat and cloacal swab	[203]
		Newcastle disease	Chicken serum	[204]
		Foot-and-mouth disease	Serum, several tissue samples	[205,206]
		Canine adenovirus	Canine serum, rectal swabs	[207]
		Brucellosis	Serum	[208]
		Gumboro disease	Poultry	[209]
	Bacteria and related infections	Bovine mastitis	Milk	[210,211,212]
		Mycobacterium bovis infection	Bovine serum, whole blood; wild boar serum	[213,214]
		Brucellosis	Dromedary camels serum	[215]
	Other infectious diseases	Bovine babesiosis	Blood	[216,217]
		Trypanosomosis	Equine serum	[218]
		Fasciolosis	Sheep serum	[219]
		Canine visceral leishmaniasis	Serum	[220,221]
		Toxoplasmosis	Cat serum	[222]
	Health status (bio)markers	Amyloid A	Horses’ serum	[223]
		Progesterone	Cattle plasma	[224]

**Table 4:** Overview of the most recent and hot LFIA applications in the environmental field.

Application Field	Target		Matrix	Reference
Environmental	Pesticides	Carbofuran and 3-hydroxy-carbofuran	Water	[225]
		Paraquat	Water	[226]
		Atrazine and acetochlor	Water	[227]
		Acetochlor and fenpropathrin	Tap water	[228]
	Pathogens	E. coli O157:H7	River water	[229]
		Human adenovirus	Wastewater	[230]
		Yersinia pestis	Suspicious white powders, aerosol samples	[231]
	Heavy metals	Lead (II)	Drinking water	[232]
	Other pollutants	Free chlorine	Aqueous soluions	[233]
		Karenia mikimotoi	Marine water	[234]
		Karlodinium veneficum	Seawater	[235]
		Microcystin-LR toxin	Water and fish	[236]
		Aflatoxin B1	Potable water	[237]
		Bisphenol A	Snow	[238]
		Norfloxacin	Tap and river water	[239]
		3-phenoxybenzoic acid	Lake water	[240]

**Table 5:** Overview of the most recent and hot LFIA applications in other application fields.

Application Field	Target		Matrix	Reference
Other	Agriculture	Banana bract mosaic virus	Banana leaf tissues	[241]
		Citrus tristeza virus	Citrus leaves	[242]
		Metalaxyl	Tobacco leaves	[243]
		Erwinia amylovora	Different plant parts	[244]
		Potato spindle tuber viroid	Plant leanves	[245]
		Dickeya solani	Potato tubers	[246]
	Forensic	Fentanyl	Human urine and serum	[247,248,249]
		Morphine, fentanyl and methamphetamine	Human urine	[250]
		Tetrahydrocannabinol	Human oral fluids	[251,252,253]
		Methamphetamine	Surface	[254]
		Prostate specific antigen and salivary amylase	Vaginal swab	[255]
		Human hemoglobin	Bloodstain	[256]
		Higenamine	Plant samples	[257]
		Hallucinogenic phenethylamines	Human Urine	[258]
	Industrial	Pantothenic acid	Pharmaceutical, food products	[259]
		Chlorogenic acid and luteoloside	Flos Lonicerae Japonicae	[260]
		Dexamethasone	Commercial facial masks	[261]
		Dihydroartemisinin and piperaquine	Commercial artemisinin-based combination therapy drugs	[262]
		Artesunate	Pharmaceutical formulation	[263]
		Artemisinin derivatives	Antimalarial drugs	[264]
		Folic acid	Orange, apple, banana, grape juice	[265]
	Other	Cotinine	Human urine	[266]

## Data Availability

The datasets generated and/or analyzed during the current study (Figure 4, Figure 5, Figure 6 and Figure 7) are available from the corresponding author on reasonable request.
